# Ultrasound-guided microwave ablation for benign thyroid nodules results in earlier and faster nodule shrinkage in patients with Hashimoto's thyroiditis than in those with normal thyroid function

**DOI:** 10.3389/fsurg.2023.1077077

**Published:** 2023-01-26

**Authors:** Yihao Chen, Weizong Liu, Chunchun Jin, Xiaohong Xu, Lifeng Xu, Jianghao Lu, Jing Zheng, Xiangmei Sun, Jiaping Feng, Sihan Chen, Zhengyi Li, Xuehao Gong

**Affiliations:** ^1^ The First Clinical Medical College, Guangdong Medical University, Zhanjiang, China; ^2^ Department of Ultrasound, Shenzhen Second People's Hospital, The First Affiliated Hospital of Shenzhen University, Shenzhen, China; ^3^ Department of Ultrasound, Affiliated Hospital of Guangdong Medical University, Zhanjiang, China

**Keywords:** thyroid neoplasms, microwave ablation, ultrasonography, threshold effect, hashimotos' thyroiditis, benign neoplasms

## Abstract

**Objectives:**

Given that the histological features of the thyroid parenchyma in patients with Hashimoto's thyroiditis (HT) differ from those of the normal thyroid gland, HT may affect the effectiveness of ultrasound-guided microwave ablation (MWA) for benign thyroid nodules (BTNs). The present study aimed to compare the effectiveness of MWA for the treatment of BTNs in patients with both BTNs and HT and those with BTNs and normal thyroid function, based on changes in the volume reduction ratio (VRR) of the BTNs.

**Methods:**

Patients who had achieved a VRR ≥50% after MWA for BTN (July 2020–June 2021), followed up for 12 months, and their data were retrospectively analyzed.

**Results:**

A total of 213 nodules were identified in 185 patients, including 167 in the “BTN” group and 46 in the “BTN + HT” group. A comparison of the fitting curves for VRR–follow-up time revealed that the VRR increased with time after MWA, although the relationship was nonlinear. Piece-wise linear regression model analysis of the threshold effect of VRR and follow-up time in the two groups indicated that the inflection point of the “BTN” group occurred at 2.1 months: VRR increased fastest within 2.1 months of MWA (rate of change: 32.9% per month; *P *< 0.001), following which the rate of change was slower and maintained at 1.0% per month (*P *= 0.006). In the “BTN + HT” group, the inflection point occurred 1.5 months after MWA, with the most significant increase occurring in this period (rate of change: 41.5% per month; *P *< 0.001), followed by a rate of 2.8% per month (*P *< 0.001) after 1.5 months.

**Conclusions:**

The relationship between VRR and follow-up time for ultrasound-guided MWA for BTN is nonlinear and exhibits a threshold effect. The current results indicated that the VRR in both groups increased before and after the inflection point, although the rate of change was greater before than after the inflection point. The inflection point occurs earlier in patients with BTN + HT than in those with BTN yet normal thyroid function, and this difference may be related to the “oven effect” involved in the development of HT.

## Introduction

Advancements in ultrasound and other imaging techniques have increased the annual rate of detection for thyroid nodules (1). Thyroid nodules are detected in approximately 70% of the general population (1); fortunately, 80%–95% of the nodules are benign (2). Nonetheless, benign thyroid nodules (BTNs) require active treatment in the following scenarios: (i) when swelling/enlargement of the nodule affects aesthetic appearance; (ii) when patients experience pressure symptoms owing to the swelling; or (iii) when the patient experiences anxiety owing to the swelling (3). Although surgery is the main treatment modality for BTN (3), open surgery is highly invasive and can lead to medically-induced hypothyroidism (4, 5). Endoscopic thyroidectomy—a minimally invasive procedure—can be performed *via* areolar, axillary, and oral vestibular approaches (6). Despite the lack of neck scarring associated with the procedure, endoscopic thyroidectomy results insignificant trauma to the subcutaneous tissue along the lumpectomy path (6). Moreover, some patients refuse to undergo surgical treatment for reasons such as surgical anxiety and unwillingness to undergo general anesthesia, which is required for this procedure (7).

Microwave ablation (MWA)—a type of thermal ablation (TA)—is a promising treatment for BTN. Ultrasound-guided (US-guided) MWA has been recommended by several international guidelines owing to its relatively low rate of complications and confirmed efficacy (3, 8, 9). Hashimoto's thyroiditis (HT) (also known as chronic lymphocytic thyroiditis) is an autoimmune thyroid disease (10) whose incidence has increased rapidly over the past 30 years, having become one of the most common thyroid diseases at present, with an incidence rate of 0.3–1.5 cases/1,000 people (11, 12). Histopathological features of HT include enlarged thyroid volume, lymphoplasmacytic infiltration, presence of fibrotic tissue, and lymphoid follicle formation (10). There is a paucity of data on the difference in efficacy of MWA treatment for BTN with or without HT. A cohort study with a short-term (1-week) follow-up reported some differences in the effectiveness of US-guided radiofrequency ablation (RFA) for the treatment of papillary thyroid microcarcinoma (PTMC) between patients with and without HT (13). Previous studies have also indicated that thermal ablation may achieve better efficacy in the treatment of liver tumors individuals with comorbid liver cirrhosis (14). This phenomenon can be attributed to the low thermal conductivity of cirrhotic liver tissue, resulting in a higher temperature of the ablated area, known as the “oven effect” (15). We postulated that the unique histopathological characteristics of the thyroid gland in cases of HT may also result in a similar “oven effect”.Consequently, this study aimed to evaluate the effectiveness of US-guided MWA for BTN with HT vs. BTN without HT, based on changes in the volume reduction ratio (VRR) of BTNs in each group.

## Materials and methods

### Patients

We retrospectively collected data for patients who underwent US-guided MWA for BTN at our institution from July 2020 to June 2021. The Ethics Committee of Shenzhen Second People's Hospital approved this study (approval number: 20220802018). This study was retrospective in design, and the requirement for specific informed consent was waived as all patients had signed the informed consent form before the MWA procedure.

The inclusion criteria were as follows:

(i) benign-appearing nodules on US subsequently, confirmed to be benign based on the results of a puncture biopsy;
(ii) unsuitability for surgical treatment or subjective refusal of the patient to undergo surgical treatment;
(iii) any one of the following four conditions in patients satisfying conditions (i) and (ii):
  (a) nodule demonstrating a notable increase in size;
  (b) symptoms associated with the nodule such as foreign body sensation, neck discomfort, or pain;
  (c) clinical manifestation of the noduleas a prominent swelling and other impacts on aesthetics, requiring treatment;
  (d) refusal of the patient to be under clinical observation due to anxiety induced by the nodule and the associated effects on daily life.

The exclusion criteria were as follows:

(i) histopathological findings indicative of malignancy or unclear pathological findings;
(ii) giant retrosternal goiter;
(iii) abnormal vocal fold function contralateral to the lesion;
(iv) coarse calcified foci within the nodule;
(v) presence of concomitant diseases, such as acute infection, severe cardiopulmonary disease, or coagulation dysfunction; and
(vi) incomplete clinical data.

The diagnosis of HT is dependent on laboratory tests supplemented by US. Serum anti-peroxidase antibody (TPOAb) and thyroid globulin antibody (TgAb) positivity is considered the most important feature of HT (10). The typical US presentation of HT is a symmetrical diffuse enlargement of the thyroid gland with marked isthmus enlargement and a hypoechoic gland with marked periphery and fine linear strong echogenicity visible in the middle, intertwined in a grid pattern with nodular hyperplasia (16). HT diagnoses were performed in accordance with these guidelines for the current study.

### Equipment and instruments

The following equipment were used for this study: (i) MyLab Twice US diagnostic instrument (Esaote, Italy), equipped with an LA533 probe (frequency, 4–13 MHz) and LA332E probe (frequency, 3–11 MHz); (ii) MTI-5A MWA therapy instrument (Nanjing Changcheng Medical Equipment Company Limited, Nanjing, China), including a microwave generator (frequency, 2450 MHz), microwave cable and XR-A1610W liquid-cooled circulation MWA needle (diameter, 16 G; length, 10 cm); and (iii) US contrast agent (Sonovue, Bracco Suisse SA, Italy), which was added to 5 ml of 0.9% saline to create a suspension for application.

### Pre-MWA assessment

First, all patients underwent pre-operative puncture biopsy of the thyroid nodules (only those who were confirmed to have benign nodules were included in present study). Second, the following pre-operative tests were performed: thyroid function-related tests including serum levels of triiodothyronine (T3), thyroxine (T4), free T3 (FT3), free T4 (FT4), and thyroid-stimulating hormone; TPOAb; TgAb; coagulation function tests; routine complete blood count; and electrocardiography. Third, laryngoscopy was performed to confirm good bilateral vocal fold movement. Finally, all patients underwent grayscale US and Color Doppler Flow Imaging (CDFI) of the thyroid; US features such as nodule location, shape, size, margin, and internal echo were recorded.

### MWA procedure

First, the patient was placed supine in the cervical hyperextension position, following which the equipment for cardiac monitoring and oxygen saturation testing was connected. Thereafter, intravenous access was established, the skin of the neck was disinfected, and sterile cavity wipes were placed. Next, pre-operative contrast-enhanced ultrasound (CEUS) was performed to determine the boundary and extent of thyroid nodules and the distribution of blood vessels, as these data were used to determine the extent of ablation. Then, 2% lidocaine hydrochloride was injected around the skin puncture site to anesthetize the thyroid envelope and surrounding tissues. For nodules with a cystic fluid component, a syringe was used to aspirate the fluid before MWA. To separate the thyroid from the common carotid artery, trachea, esophageal space, and the posterior thyroid space (the recurrent laryngeal nerve zone), a 0.9% saline injection was used to form a “fluid isolation” area, thereby protecting these important structures from thermal damage. Next, the output power of the MWA instrument was set at 30 W, and the liquid-cooled MWA electrode needle was placed inside the target nodule *via* percutaneous puncture under US guidance. The target nodule was then ablated layer by layer using the “moving-shot technique” until it was completely covered by the strong echogenic vaporization zone (3, 17), and CDFI demonstrated no blood flow signal in the nodule. Immediately after MWA, CEUS was performed to observe the ablated area and to determine the completion of ablation, and in case of residual enhancement area, re-ablation was performed until the nodule was completely inactivated. Intermittent compression was performed with an ice pack at the neck puncture site for 6 h after MWA to avoid bleeding and thermal injury.

### Post-MWA follow-up

Patients were required to undergo regular US of the thyroid after MWA to assess the effectiveness of the treatment, with the generally recommended follow-up dates being 1, 3, 6, and 12 months after MWA. Nodule volume was determined according to the following formula (8):

V(ml)=length×width×depth×0.524.

The VRR refers to the percentage decrease in nodule volume between initial imaging presentation and the current follow-up point.In this study, a VRR of ≥50% was considered to indicate the effectiveness of the treatment (8).


$$\mathrm{VRR}\left(\%\right)=\frac{\left(initial\;volume\;-\;follow-up\;volume\right)}{initial\;volume}\;\times \;100\%.$$

We used the Society of Interventional Radiology (SIR) standardized grading system to grade the severity of adverse events associated with MWA (18). The SIR classified adverse events into three grades: side effects, minor complications, and major complications (8, 18).

### Data collection

The following data were collected in this study: (i) patient demographics, including name, sex, and age; (ii) thyroid function values (presence or absence of HT); (iii) US characteristics of BTN (number, location, size, shape, margin, internal echoes), and initial volume calculated from the three diameter lines measured on US; and (iv) nodule volume and VRR at each follow-up point.

Owing to the epidemic prevention policies, some patients could not be followed up at exactly the 1, 3, 6, and 12 months-mark after MWA during the COVID-19 pandemic. Therefore, we collected the actual follow-up date (for example, 1, 5, 8, and 12 months after MWA) and the volume of nodules and calculated the corresponding VRR for each patient. According to the purpose of this study, patients with a VRR ≥50% during the 12-month follow-up period were included in this study.

### Statistical analysis

The statistical software used in this study was SPSS (version 20.0; SPSS, Chicago, IL, USA), R (http://www.R-project.org), and EmpowerStats software (www.empowerstats.com, X&Y solutions, Inc. Boston MA). Categorical variables were analyzed using the Chi-Square test. The measurement data are expressed as mean ± standard deviation. In case of normally distributed data and similar variance, *t*-test were used. For data that were not normally distributed, Mann–Whitney's *U* test were used.

Changes in VRR over time were analyzed using a generalized additive model to generate a fitting curve,and threshold effect analysis was used to determine thethreshold (i.e., inflection point) for VRR over time. The nonlinear relationship between VRR and follow-up time was confirmed based on the fitting curve and the results of the likelihood ratio test, following which a piece-wise linear regression model was used to calculate the threshold values (19, 20). *P* values less than 0.05 were considered statistically significant.

## Results

### Patient selection

**Figure 1:**
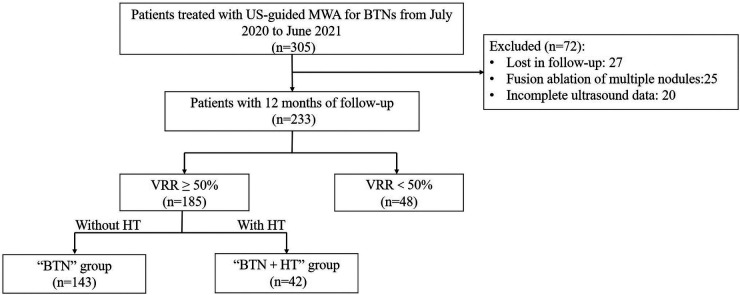
Flow chart of patient selection for this study. BTN: benign thyroid nodule; HT, Hashimoto's thyroiditis; VRR, volume reduction ratio; MWA, microwave ablation.

During the study period, 305 patients underwent MWA for the treatment of BTNs, 72 of whom were excluded because of reasons such as loss to follow-up, incomplete US data (US sections were incomplete or non-standard, making volume calculation difficult), and fusion ablation, as listed in Figure 1, which illustrates the flow chart the inclusion process. Another 48 patients were excluded due to a VRR of <50%. Finally, 185 patients (213 nodules) were selected and divided into two groups: a “BTN” group comprising patients with normal thyroid function (143 patients with 167 nodules) and a “BTN + HT” group (42 patients with 46 nodules).

### Baseline data

Table 1 demonstrates the demographic, clinical, and sonographic data for patients included in this study. The proportion of females patients was significantly higher in the “BTN + HT” group than in the “BTN” group (95.24% *vs.* 80.42%, *P *= 0.027), although there was no significant difference in age between the two groups (*P *= 0.859). Moreover, there were no significant differences between the two groups in terms of US characteristics such as location, shape, margin, and echogenicity of the nodules (*P *> 0.05). In the “BTN” and “BTN + HT” groups, the maximum diameters of the nodules were 29.74 ± 10.79 mm and 27.96 ± 10.44 mm, respectively; the respective values for nodule volume were 7.31 ± 8.19 ml and 5.89 ± 5.50 ml, while those for ablation times were 358.67 ± 276.26 s and 277.48 ± 197.30 s.There were no significant differences in these three parameters between the groups (*P* for all three parameters >0.05). In terms of nodule location, 53.29% (89/167) of the nodules in the “BTN” group and 60.87% (28/46) in the “BTN + HT” group were located <2 mm from the trachea (i.e., adjacent to the trachea) (*P *> 0.05); in contrast, 46.71% (78/167) and 45.65% (21/46) of the nodules in the “BTN” and “BTN + HT” groups, respectively, were located adjacent to the recurrent laryngeal nerve (*P *> 0.05).

**Table 1:** Comparison of baseline information between the “BTN” and “BTN + HT” groups.

	“BTN” group	“BTN + HT” group	Total	*P*
**Patients (*n*)**	143	42	185	
**Nodules (*n*)**	167	46	213	
**Age (years)**	41.83 ± 11.85	42.19 ± 11.20	42.53 ± 11.74	0.859
**Sex**				0.027
Male (n)	28	2	30	
Female (n)	115	40	155	
**Location**				0.489
Left Lobe (*n*)	72	21	93	
Right Lobe (*n*)	90	25	115	
Isthmus (*n*)	5	0	5	
**Shape**				0.386
Regular (*n*)	132	39	171	
Irregular (*n*)	35	7	42	
**Margin**				0.314
Defined (*n*)	138	35	173	
Ill-Defined (*n*)	29	11	40	
**Echo**				0.663
Hypoechoic (*n*)	111	26	137	
Isoechoic (*n*)	33	12	45	
Hyperechoic (*n*)	8	3	11	
Mixed Echoic (*n*)	15	5	20	
**Adjacent to the trachea**				0.360
Yes (*n*)	89	28	117	
No (*n*)	78	18	96	
**Adjacent to the recurrent laryngeal nerve**				0.899
Yes (*n*)	78	21	117	
No (*n*)	89	25	96	
**Maximum diameter (mm)**	29.74 ± 10.79	27.96 ± 10.44	29.35 ± 10.72	0.320
**Volume (ml)**	7.31 ± 8.19	5.89 ± 5.50	7.01 ± 7.70	0.453
**Ablation time (s)**	358.67 ± 276.26	277.48 ± 197.30	341.14 ± 262.95	0.079

Data are expressed as number (*n*) where indicated.

BTN, benign thyroid nodule; HT, Hashimoto's thyroiditis.

### VRR in the “BTN” and “BTN + HT” groups

**Figure 2:**
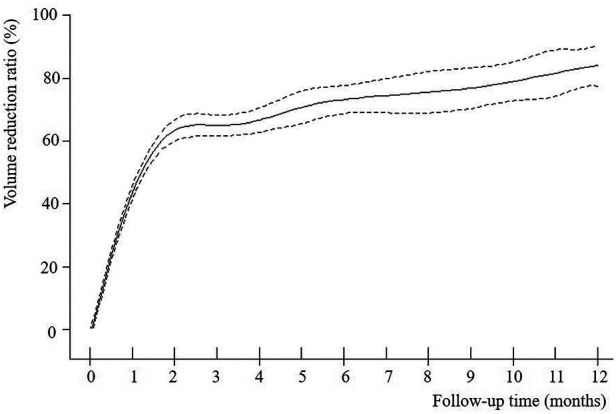
VRR-Time fitting curve for “BTN” group. BTN, benign thyroid nodule; VRR, volume reduction ratio.

**Figure 3:**
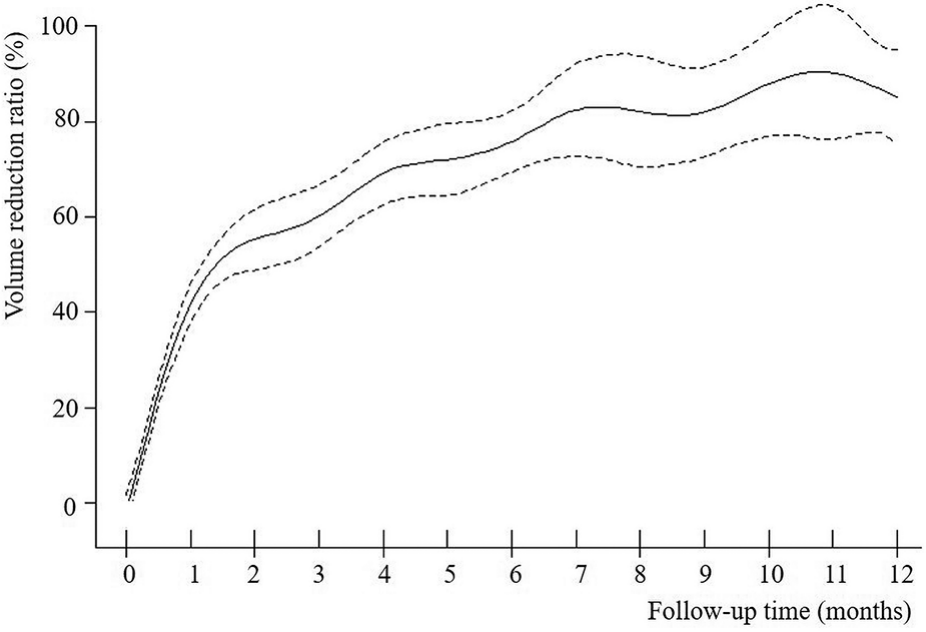
VRR-Time fitting curve for “BTN + HT” group. BTN, benign thyroid nodule; HT, Hashimoto's thyroiditis; VRR, volume reduction ratio.

**Figure 4:**
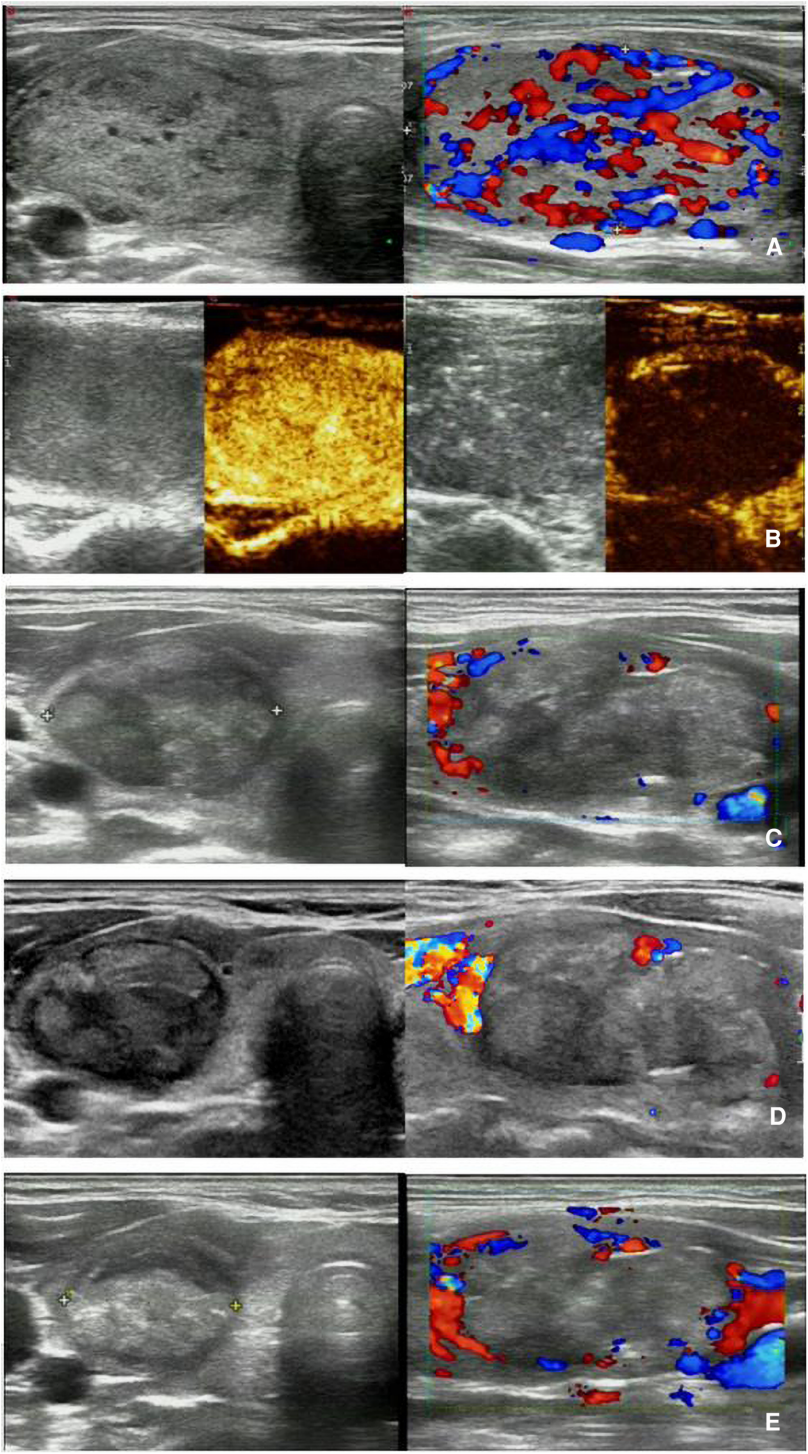
MWA-treated BTN with HT in a 37-year-old woman. (**A**) Pre-MWA transverse grayscale ultrasound (left) and longitudinal CDFI (right) demonstrate a nodule with regular shape and clear margin, abundant blood flow, size of 48 × 31 × 33 mm, volume of approximately 17.93 ml, and pathologically confirmed as BTN by needle biopsy; (**B**) Pre-MWA CEUS (left) shows the nodule with uniform hyperenhancement, and post-MWA CEUS (right) shows the nodule without enhancement; C-E. Post-MWA at 1 month (**C**), 3 months (**D**), and 6 (**E**) months after MWA; transverse grayscale ultrasound (left) and longitudinal CDFI (right) shows that the nodules gradually decreased size, with sizes of 40 × 26 × 20 mm, 34 × 22 × 18 mm, and 32 × 18 × 16 mm, respectively, volumes of approximately 10.90 ml, 7.06 ml, and 4.83 ml, respectively, and VRR of 39.21%, 60.62%, and 73.06% respectively. MWA, microwave ablation; BTN, benign thyroid nodule; HT, Hashimoto's thyroiditis; VRR, volume reduction ratio; CDFI, Color Doppler Flow Imaging; CEUS, contrast-enhanced ultrasound.

The VRR of nodules in both groups increased with time after MWA and demonstrated nonlinear relationship (Figures 2–4). Piece-wise linear regression analysis of the threshold effect of follow-up time on VRR indicated that the inflection points of the “BTN” and “BTN + HT” groups were at 2.1 months and 1.5 months after MWA, respectively (Table 2). As demonstrated in Figure 2, Table 2, the fastest increase in VRR for the “BTN” group occurred within the first 2.1 months after MWA (rate of change: 32.9% per month; *P *< 0.001), following which the rate of change decreased to 1.0% per month (*P *= 0.006). Figure 3, Table 2 also show that the fastest increase in the rate of change for the “BTN + HT” group occurred within the first 1.5 months after MWA (rate of change: 41.5% per month; *P *< 0.001), following which the rate of change decreased to 2.8% per month (*P *< 0.001).

**Table 2:** Threshold effect for the relationship of follow-up time with VRR using piece-wise linear regression.

	*β*	95% *CI*	*P*
VRR in “BTN” group			
Time ≤ 2.1 months	32.9	(30.9, 34.9)	<0.001
Time > 2.1 months	1.0	(0.3, 1.7)	0.006
VRR in “BTN + HR” group			
Time ≤ 1.5 months	41.5	(37.4, 45.6)	<0.001
Time > 1.5 months	2.8	(2.0, 3.6)	<0.001

BTN, benign thyroid nodule; HT, Hashimoto's thyroiditis; VRR, volume reduction ratio; CI, confidence interval.

### Adverse events

Major complications—as defined by SIR—did not occur. The following cases of minor complications were recorded: two patients in the “BTN” group had transient hoarseness after MWA, both of whom recovered spontaneously after 1 week and after 2 months without requiring any additional treatment; one patient in the “BTN” group experienced post-operative neck swelling after MWA and recovered after 1 month. Regarding side effects, six patients in the “BTN” group and two patients in the “BTN + HT” group developed obvious neck pain and involvement of the face, teeth, and ears during or after MWA, and all symptoms disappeared completely within 1 week of the procedure.

## Discussion

In the present study, we compared the effectiveness of ultrasound-guided MWA for the treatment of BTNs in patients with both BTNs and HT and those with BTNs and normal thyroid function, based on changes in the VRR. Our findings indicated that the relationship between VRR and follow-up time for US-guided MWA for BTN was nonlinear. An analysis of the threshold effect of follow-up time on VRR, indicated that the inflection points of the “BTN” and “BTN + HT” groups occurred at 2.1 months and 1.5 months after MWA, respectively.

Surgical procedures are currently the main clinical treatment for BTN (3). TA techniques such as RFA, MWA, and laser ablation (LA) have shown promise as alternatives to the surgical treatment of BTN. These techniques can all be performed by inserting ablation electrodes into the target tissues under the guidance of US and other imaging equipment. Although these procedures generate heat through different forms of physical energy, each is designed to induce coagulative necrosis of tissues at high temperatures, and the necrotic tissues are eventually cleared and absorbed by the body's immune system, resulting in physical local inactivation to achieve treatment success (21). Compared with that of RFA and LA, the effectiveness of MWA is not affected by tissue carbonization or current conduction and has the advantages of more uniform heat distribution, reduced heat loss, larger ablation range, and shorter ablation time (22). US-guided MWA has the advantages of high safety, precise efficacy, preservation of thyroid function, reproducibility, minimal trauma, and rapid recovery, and it can be performed on an outpatient basis under local anesthesia and has been established as a clinicallyvalid safe and effective method for the treatment of BTN (23).

HT is a slowly progressive disease with histopathological manifestations comprising gradual destruction and atrophy of normal thyroid follicles, lymphocytic infiltration and hyperplasia, and fibrous tissue hyperplasia forming vitelliform changes. In the mid to late stages of the disease, the fibrous tissue proliferates, the gland hardens, and single or multiple nodules are formed (10). Given that the histological features of the thyroid parenchyma in patients with HT differ from those of the normal thyroid gland, HT may affect the effectiveness of MWA for thyroid nodules. At present, there no studies have reported whether temporal trends in the VRR after MWA differ according to the presence of HT in patients with BTNs. To address this issue, we investigated changes in the VRR in these two groups of patients treated at our institution.

In the present study, the proportion of women was significantly higher in the “BTN + HT” group than in the “BTN” group (*P *= 0.027), which is consistent with the high female prevalence of HT of 5%–10% (24, 25). A previous report noted that nodules adjacent to the trachea and the recurrent laryngeal nerve may demonstrate incomplete ablation (26); in the present study, we observed no difference in the number of nodules adjacent to the “danger zone” between the groups. The above comparison results indicate that the baseline information of the two groups of patients was similar and comparable except for sex. The current findings indicated that the VRR of both groups gradually increased with time in both groups in a nonlinear fashion, and that the relationship exhibited a threshold effect. The inflection point in the “BTN + HT” group was observed at 1.5 months, which is slightly earlier than the inflection point of 2.1 months in the “BTN” group.

Most published studies exploring MWA for BTN have used fixed time points for follow up, commonly recording changes in volume and VRR at 1, 3, 6, and 12 months after ablation (23, 27, 28). However, in actual clinical practice, follow-up times are influenced by poor patient compliance and insufficient knowledge of the disease. Moreover, considering the influence of the COVID-19 pandemic and our epidemic prevention policy (29), a significant proportion of patients in this retrospective study were unable to visit our healthcare facility for follow-up at the prescribed time points. Nevertheless, in this study, using the data from clinical practice, we dutifully recorded the MWA follow-up times, the nodule volumes, and VRR data for the included patients.

We used a piece-wise linear regression model to analyze the threshold effect and determine the inflection point for VRR in each group. The inflection point reflects achange in the acceleration of changes in nodule size. Prior to the inflection point, rates of change are rapid. After the inflection point, the rate begins to decrease, although changes in size can still be observed. As mentioned previously, the inflection point occurred earlier in the “BTN + HT” group than in the “BTN” group. We suspect that histological changes in the thyroid parenchyma caused by HT influenced the efficacy of MWA for BTN, given that patients with HT concomitant with BTN exhibited earlier and faster nodule shrinkage in the early post-MWA period. Specifically, these differences may be related to differences in the thermal conductivity of the thyroid tissue between the two groups. Liu et al. (15) found that the thermal conductivity of background tissue is important for tumor heating based on *in vitro* models. When the environmental thermal conductivity around the tumor is low, the heat of ablation will be concentrated inside the tumor. In essence, the sclerotic tissue surrounding the tumor may act as an insulator, increasing the heat inside the tumor and preventing heat loss to the outside, which is known as the “oven effect” (15). An *in vitro* study also observed that heat is more likely to be deposited inside liver tumors surrounded by cirrhosis (14). Histopathologically, HT is characterized by the destruction of normal thyroid follicular cells due to lymphocytic infiltration, which is responsible for HT-induced hypothyroidism (10). Simultaneously, fibrous tissue hyperplasia causes gland atrophy, fibrosis, and hardening. Given these pathological changes, the thyroid tissue of HT may create an “oven effect” similar to that produced by cirrhotic tissue in the liver. During MWA treatment, heat may therefore accumulate within BTNs, leading to rapid coagulation necrosis. Owing to the diffuse chronic inflammatory cells present in the thyroid gland in patients with HT, the autoimmune system is activated more quickly, and necrotic substances are phagocytosed and eliminated (30). This may explain the faster rate of shrinkage for nodules in the “BTN + HT” group.

In clinical practice, we often encounter patients with HT who ask whether they can receive MWA treatment for their BTNs, and the corresponding evidence was previously unavailable to inform our answers to this question. To the best of our knowledge, few studies have investigated potential differences in the effectiveness of MWA for BTN between patientswith and without HT. However, some studies have investigated the effectiveness of RFA for PTMC in patients with or without HT (13, 31). Interestingly, these two studies reached inconsistent conclusions and had certain limitations. Zhang et al. (31) analyzed data for 30 patients with PTMC and 30 patients PTMC + HT.The authors concluded that RFA was effective in patients with PTMC + HT, and that its efficacy and safety were similar to those for patients with PTMC without HT through 1–18 months of follow-up. While the sample size in this study was considerably small, the follow-up time was adequate. The other study was a large cohort study of 797 nodules (573 *vs.* 224 nodules in the “PTMC” group *vs.* “PTMC + HT” group) that focused on the effect of RFA for early-stage PTMC on the extent of the ablation zone in those with HT (13). After 1 week of RFA treatment, the size of PTMCs was smaller in patients with HT than in thosewith a healthy thyroid. Despite thelarge size of the cohort, a 1-week follow-up is too short for evaluating the effect of RFA for PTMC, as the ablation area may change after the inflammatory edema period has elapsed. Given the lack of evidence in this area, we conducted the current retrospective study to investigate whether the effects of MWA differ between patients with BTN and those with BTN + HT. The preliminary finding that the inflection point occurred 15 days earlier in the BTN + HT group without increases in the rate or severity of adverse events may help to allay patient concerns.

In terms of adverse events, two patients experienced temporary hoarseness owing to thermal damage of the recurrent laryngeal nerve, and this may be attributed to the close proximity of the nerve to the nodules and to the loss of isolation fluid during the ablation process without timely replenishment. During MWA, radiating pain in the face, ears, and teeth may occur due to heat transfer during ablation of the nodular envelope near the superficial thyroid gland, although this pain generally resolves spontaneously at the end of the ablation procedure (32). Overall, there was no difference or specificity in the incidence of complications between the two groups in the current study.

The present study had some limitations. First, this was a retrospective study with unavoidable patient selection bias, and the greater frequency of female patients in the “BTN + HT” group may have had certain effects on the results. Second, this study used a fixed ablation power of 30 W. We were unable to compare treatment effects and thresholds for different ablation powers, which can cause differences in the degree of carbonation, which in turn affects the VRR. Third, HT is a chronic progressive disease, and the histopathology of thyroid glands varies at different time periods. Therefore, future large-scale studies should aim to compare differences in the effectiveness of on MWA for BTN concomitant with HT at different time periods, are warranted.

## Conclusion

The VRR exhibits a nonlinear relationship with follow-up time during US-guided MWA for BTNs, for which a threshold effect can be observed. The current results indicated that the VRR in both groups increased before and after the inflection point, although the rate of change was greater before than after the inflection point. The earlier inflection point observed in the “BTN + HT” group may be related to the “oven effect” of affected tissue in patients with HT; however, additional studies are required to determine the exact mechanism underlying these differences.

## Data Availability

The raw data supporting the conclusions of this article will be made available by the authors, without undue reservation.
